# Risk factors for anastomotic leakage and its impact on survival outcomes in radical multivisceral surgery for advanced ovarian cancer: an AGO-OVAR.OP3/LION exploratory analysis

**DOI:** 10.1097/JS9.0000000000002306

**Published:** 2025-02-24

**Authors:** Fabian Trillsch, Bastian Czogalla, Sven Mahner, Verena Loidl, Alexander Reuss, Andreas du Bois, Jalid Sehouli, Francesco Raspagliesi, Werner Meier, David Cibula, Alexander Mustea, Ingo B. Runnebaum, Barbara Schmalfeldt, Giovanni Aletti, Rainer Kimmig, Giovanni Scambia, Felix Hilpert, Annette Hasenburg, Uwe Wagner, Philipp Harter

**Affiliations:** a Department of Obstetrics and Gynecology, LMU University Hospital, LMU Munich, Munich, Germany; b Faculty of Medicine, Institute for Medical Information Processing, Biometry, and Epidemiology – IBE, LMU Munich, Munich, Germany; c Coordinating Center for Clinical Trials, Philipps University Marburg, Marburg, Germany; d Department of Gynecology and Gynecologic Oncology, Ev. Kliniken Essen-Mitte, Essen, Germany; e Department of Gynecology, Charité-Universitätsmedizin Berlin, Berlin, Germany; f Gynecologic Oncology Unit Fondazione IRCCS Istituto Nazionale Tumori, Milan, Italy; g Department of Obstetrics and Gynecology, Heinrich-Heine-University Düsseldorf, Germany; h Department of Obstetrics, Gynaecology and Neonatology, General University Hospital in Prague, First Faculty of Medicine, Charles University, Czech Republic; i Department of Gynecology and Gynecological Oncology, Bonn University Hospital, Bonn, Germany; j Department of Gynecology and Reproductive Medicine and Center for Gynecologic Oncology, Jena University Hospital, Jena, Germany; k Department of Gynecology, University Medical Center Hamburg-Eppendorf, Hamburg, Germany; l Department of Gynecologic Oncology, European Institute of Oncology, University of Milan, Italy; m Department of Gynecology and Obstetrics, University of Duisburg-Essen, Essen, Germany; n Fondazione Policlinico Universitario A. Gemelli IRCCS, Università Cattolica del S. Cuore Rome, Rome, Italy; o Oncologic Medical Center at the Jerusalem Hospital Hamburg, Hamburg, Germany; p University Medical Center Mainz, Department of Gynecology and Obstetrics, Mainz, Germany; q Department of Gynecology and Obstetrics, University Hospital Giessen and Marburg, Marburg, Germany

**Keywords:** anastomotic leakage, multivisceral surgery, ovarian cancer, risk factors, stoma formation, survival

## Abstract

**Background:**

Anastomotic leakage is a significant complication following bowel resection in cytoreductive surgery for ovarian cancer. Previous studies have highlighted the detrimental effects of anastomotic leakage on patients’ postoperative course. However, there is still a lack of precise identification of the high-risk population and established strategies for preventing its occurrence.

**Materials and methods:**

Patients who underwent bowel resection within the surgical phase III trial AGO-OVAR.OP3/LION investigating the impact of systematic pelvic and paraaortic lymphadenectomy in cytoreductive surgery for primary ovarian cancer were included in this analysis. All patients in the AGO-OVAR.OP3/LION trial had undergone complete cytoreduction with no macroscopic residual disease. We analyzed the occurrence of anastomotic leakage regarding surgical procedure (non-lymphadenectomy vs. lymphadenectomy and non-stoma vs. stoma) using the Fisher test. Risk factors for anastomotic leakage and its prognostic impact on survival were analyzed.

**Results:**

Overall rate of anastomotic leakage was 7.1%. Notably, the Non-lymphadenectomy subgroup had a lower anastomotic leakage rate of 3.0% compared to the lymphadenectomy subgroup (11.2%, *P* = 0.005). The use of protective stoma placement resulted in an anastomotic leakage rate of 5.5% regardless of lymphadenectomy compared to the Non-Stoma subgroup (7.5%, *P* = 0.78). Increased blood loss (odds ratio [OR] 1.04 per 100cc, 95% confidence interval [CI] 1.0001–1.09) and lymphadenectomy (OR 3.67, 95% CI 1.41–11.40) were associated with a higher risk of anastomotic leakage. Although anastomotic leakage demonstrated a numerical detrimental impact on median progression-free survival (PFS) (18 months with anastomotic leakage vs. 19 months with Non-anastomotic leakage, hazard ratio [HR] 0.86; 95% CI 0.5 to 1.4, *P* = 0.53) and median overall survival (OS) (31 months with anastomotic leakage vs. 58 months with Non-anastomotic leakage, HR 0.69; 95% CI 0.4 to 1.2, *P* = 0.17), the differences were not statistically significant.

**Conclusion:**

Anastomotic leakage rates were lower in the Non-lymphadenectomy arm, the current standard of care. Blood loss and lymphadenectomy, as surrogate markers for extensive surgery, were associated with increased risk for anastomotic leakage. These findings highlight the importance of strategies to reduce surgical complexity and perioperative risk to improve clinical outcomes.

## Introduction

Ovarian cancer is the most lethal gynecologic malignancy and is mostly diagnosed in advanced stage disease, resulting in a poor prognosis for patients despite recent advances in systemic treatment^[1,2]^. Radical debulking surgery aiming at complete cytoreduction to microscopic disease is considered the most important prognostic factor for survival^[3-5]^. Bowel surgery is frequently performed as a standard procedure in multivisceral surgery to achieve complete tumor resection. However, it is associated with a significant overall risk for life-threatening complications and specifically anastomotic leakage^[6-9]^. Reported incidences in ovarian cancer surgery range from 1.2% to 16.9%^[6,9-21]^. Anastomotic leakage not only leads to prolonged hospitalization but also causes delays in initiating chemotherapy which might have negative impact on prognosis^[6,8-12,14-17,20]^. In contrast to colorectal cancer, reliable risk factors for anastomotic leakage in ovarian cancer surgery have not yet been identified. Retrospective analyses suggest risk factors as age, poor nutritional status, low albumin level, bevacizumab treatment, previous pelvic irradiation, the distance of the anastomosis from the anal verge, multiple bowel resections and handsewn anastomosis^[8-10,21-25]^. However, validation of these mostly monocentric findings from retrospective analyses is pending and investigation in larger cohorts from prospective surgical clinical trials in ovarian cancer has not yet been performed.

As possible protective measures to reduce the incidence and consequences of anastomotic leakage, stoma placement has been proposed in the past^[26-29]^. However, studies suggest that protective stoma placement does not specifically reduce rates of anastomotic leakage but mainly decreases the rates of overall complications such as sepsis and the need for re-laparotomy^[17]^. Specific effects of stoma formation in ovarian cancer surgery have not been demonstrated so far.

AGO-OVAR.OP3/LION was a randomized controlled phase III trial investigating the impact of systematic lymphadenectomy in advanced ovarian cancer patients who had undergone complete cytoreduction with no macroscopic residual disease and were intraoperatively confirmed to have non-suspicious lymph nodes^[30]^. For this cohort, no impact on progression-free (PFS) or overall (OS) survival was identified but higher surgical burden and morbidity have been described so that this study subsequently defined the standard of care in ovarian cancer surgery with omission of systematic lymphadenectomy in advanced stage disease. In this prospective surgical trial, all patients were treated in quality assured and centrally certified experienced surgical centers for gynecologic oncology. The included study population consisted of patients experiencing the highest benefit from surgery through complete cytoreduction and had a median OS of 69.2 months in the Non-lymphadenectomy group^[30]^. This exploratory subgroup analysis evaluated the impact and risk factors of anastomotic leakage.

## Material and methods

### Patients and centers

All analyses of the present exploratory subgroup were carried out on data of patients undergoing bowel surgery within the AGO-OVAR.OP3/LION trial. In brief, AGO-OVAR.OP3/LION trial intraoperatively randomized 647 patients with advanced ovarian cancer following complete cytoreduction with non-suspicious lymph nodes to either undergo systematic pelvic and paraaortic lymphadenectomy or not. Eligible patients for this clinical trial met the following criteria: confirmed histological diagnosis of advanced epithelial ovarian cancer, ranging from Fédération Internationale de Gynécologie et d’Obstétrique (FIGO) stage IIB to IV. In cases of FIGO stage IIB to III disease, the cancer was confined to the peritoneal cavity, while patients with metastases outside the peritoneal cavity (FIGO stage IV) could be included if metastases in the pleura, liver, spleen, or abdominal wall were considered to be resectable. Additional inclusion criteria were age between 18 and 75 years, option of complete cyotreduction following clinical evaluation, and good Eastern Cooperative Oncology Group (ECOG) performance status score of 0 or 1. Intraoperative randomization within the trial was only performed if surgical cytoreduction to microscopic disease was achieved and no suspicious lymph nodes were found following evaluation of the retroperitoneal space up to the renal vein.

All surgeries were performed by experienced gynecologic oncologists in high-volume centers adhering to standardized surgical protocols. An independent review of anonymized surgical and pathological reports was conducted, detailing systematic pelvic and paraaortic lymphadenectomy procedures performed within the preceding 12 months. To qualify, centers were required to demonstrate that at least 12 operations conducted in the prior year met the predefined quality criteria outlined in the original trial protocol (chapter 6.4)^[30]^.

**HIGHLIGHTS**

- Overall rates for anastomotic leakage were low in highly specialized centers for gynecological oncology radical surgery performing the AGO-OVAR.OP3/LION trial. No significant protective effects for stoma placement in case of bowel surgery could be demonstrated in the analyzed cohort.
- Although anastomotic leakage showed a numerical trend towards worse outcomes, no statistically significant impact was observed, highlighting the need for further investigation and strategies to evaluate this perioperative complication.
- Blood loss and systematic pelvic and paraaortic lymphadenectomy, as surrogate markers for extensive surgery, are associated with higher anastomotic leakage rates.

Of the original 647 patients randomized in the trial, 311 patients were excluded from this exploratory subgroup analysis as they did not undergo bowel surgery. Only patients with confirmed bowel resections and complete clinical follow-up were included in this analysis. Patients were excluded if they did not require bowel surgery or if relevant postoperative data were unavailable.

Anastomotic leakage was assessed during the postoperative period using clinical signs (e.g., fever, peritonitis), laboratory markers (e.g., elevated inflammatory markers), and imaging studies (e.g., contrast-enhanced CT) when clinically indicated. In specific circumstances, endoscopic evaluation may be performed to confirm or rule out clinical suspicion before proceeding with re-laparotomy.

Written informed consent was required for participation in the trial. A more detailed description including the trial protocol and statistical analysis plan can be found in original publication^[30]^. The analysis has been reported in line with the CONSORT criteria^[31]^.

### Statistics

Categorical variables were assessed with Chi-Squared or Fisher exact tests as well as continuous variables with Mann–Whitney *U* test, as appropriate.

Potential prognostic impact of anastomotic leakage on survival probabilities was analyzed by Kaplan–Meier method and compared using the log-rank test. OS was calculated from date of randomization to the date of death, PFS from randomization to date of first disease progression, or date of death (whichever occurred first). Survival time of patients alive at the last follow-up was censored. Risk factors for anastomotic leakage were evaluated by calculating odds ratio (OR) with 95% profile-likelihood confidence interval (CI) in univariate and multivariate logistic regression models. Stepwise variables selection algorithm using the Akaike´s Information Criteria were used for identification of the final logistic regression model. The logistic regression model was built using a stepwise selection approach. The initial null model included only the intercept (AI ~ 1), and the full model included all candidate variables of interest. The stepwise selection was performed bidirectionally (both forward and backward) using the step() function in R, which iteratively evaluates the inclusion or exclusion of variables based on Akaike´s Information Criterion. *P* values presented two-tailed, and *P* values <0.05 were considered statistically significant. Due to the exploratory character of this analysis, no *P* value adjustment was applied. Thus, the results have to be interpreted descriptively. All analyses were performed using the statistical programming software packages R version 4.2.2 (R Corporation for Statistical Computing)^[32,33]^.

## Results

### Patients’ characteristics

In this data set of 336 patients undergoing bowel surgery within the AGO-OVAR.OP3/LION trial, 24 patients were diagnosed with anastomotic leakage during postoperative clinical follow-up within 60 days after surgery. In Table 1, detailed demographic and clinicopathological characteristics of patients experiencing anastomotic leakage are opposed to patients without anastomotic leakage. Possible risk factors as age, BMI, FIGO stage, surgery duration and type of bowel resection did not significantly differ between subgroups. In addition, placement of a protective stoma was found to be more frequent in the Non-anastomotic leakage group with 16.7% compared to 12.5% in the anastomotic leakage group, albeit not significantly different (*P* = 0.81).

**Table 1:** Clinical characteristics of all patients undergoing bowel surgery

Clinical characteristics	Anastomotic leakage	No Anastomotic leakage	Overall Population	*P*-value
	(*N* = 24)	(*N* = 312)	(*N* = 336)	
Median age (range) – yr	63 (47–74)	62 (21–79)	62 (21–78)	0.32
Median BMI (range) – kg/m^2^	24.3 (17.9–32.7)	24.5 (16.4–55.0)	24.5 (16.38–55.0)	0.55
ECOG performance status score – no. (%)				
0	20 (83.3)	260 (83.3)	280 (83.4)	1.00
1	4 (16.7)	52 (16.7)	56 (16.7)	
ASA physical status classification system – no. (%)				
1	11 (45.8)	103 (33.0)	114 (33.9)	0.27
2	9 (37.5)	170 (54.5)	179 (53.3)	
3	4 (16.7)	39 (12.5)	43 (12.8)	
Median CA-125 level before surgery (range) – U/ml	342 (11–3310)	450 (9–28 850)	447 (9–28 850)	0.58
Final histologic diagnosis – no. (%)				
Ovarian, fallopian tube, or peritoneal cancer	23 (95.8)	290 (92.9)	313 (93.2)	0.91
Other diagnosis, including borderline tumor	1 (4.2)	22 (7.1)	23 (6.8)	
Final FIGO stage – no. (%)				
IA/IB/IC/IIA	0 (0)	5 (1.6)	5 (1.5)	0.42
IIB/IIC/IIIA	0 (0)	20 (6.4)	20 (6.0)	
IIIB/IIIC/IV	24 (100)	279 (89.4)	303 (90.2)	
Median surgery duration (range) – min	382 (210–720)	360 (97–720)	360 (97–720)	0.09
Ascites > 500 cc – no. (%)	13 (54.2)	139 (44.6)	152 (45.2)	0.48
Median blood loss – per 100cc (range)	1000 (0–6000)	700 (0–4800)	800 (0–6000)	**0.007**
Small bowel resection – no. (%)	5 (20.8)	89 (28.5)	94 (28.0)	0.57
Large bowel resection – no. (%)	24 (100.0)	292 (93.6)	316 (94.0)	0.41
Stomach resection – no. (%)	1 (4.2)	21 (6.7)	22 (6.5)	0.95
Stoma placement – no. (%)				
none	21 (87.5)	260 (83.3)	281 (83.6)	0.81
temporary/permanent	3 (12.5)	52 (16.7)	55 (16.4)	
Splenectomy – no. (%)	6 (25.0)	84 (26.9)	90 (26.8)	1.00
Lymphadenectomy – no. (%)	19 (79.2)	150 (48.1)	169 (50.3)	**0.006**

yr: years, SD: standard deviation, no.: number, cc: cubic centimeter

In contrast, significantly higher volume of blood loss was found for patients with anastomotic leakage compared to patients without anastomotic leakage (median [range]: 1000 cc [0–6000] vs. 700 cc [0–4800]), *P* = 0.007). Lymphadenectomy was significantly more common with 79.2% in the anastomotic leakage group compared to 48.1% in the Non-anastomotic leakage group (*P* = 0.006).

### Anastomotic leakage rate and stoma placement

A total of 24 out of all 336 patients who underwent bowel surgery were diagnosed with anastomotic leakage, resulting in an overall anastomotic leakage rate of 7.1%. In the entire AGO-OVAR.OP3/LION trial cohort of 647 patients, including those who did not undergo bowel surgery, the anastomotic leakage rate was 3.7%. Among patients undergoing lymphadenectomy 19 of 169 patients (11.2%) had anastomotic leakage compared to 5 of 167 patients without lymphadenectomy (3.0%, *P* = 0.005).

Overall, the majority of 281 patients (83.6%) underwent radical surgery including bowel resection but did not receive a protective stoma and were found to have an anastomotic leakage rate of 7.5% (21 out of 281 patients). Among the 55 patients with stoma placement, the anastomotic leakage rate was 5.5% (3 out of 55 patients), and this difference was not statistically significant (*P* = 0.78).

Within the 167 patients randomized to the Non-lymphadenectomy arm, 5 of the 145 patients (3.5%) without a stoma experienced an anastomotic leakage, while none of the 22 patients with stoma was diagnosed with anastomotic leakage (*P* = 1.0) (Table 2).

**Table 2:** Number of patients with anastomotic leakage and rate according to the surgical procedures lymphadenectomy and stoma placement

Stoma placement	anastomotic leakage (rate) according to surgical procedure		
	All patients	lymphadenectomy	Non-lymphadenectomy
	(*n* = 336)	(*n* = 169)	(*n* = 167)
Regardless	24/336 (7.1%)	19/169 (11.2%)	5/167 (3.0%)
No	21/281 (7.5%)	16/136 (11.8%)	5/145 (3.5%)
Yes	3/55 (5.5%)	3/33 (9.1%)	0/22 (0.0%)
p-value*	0.78	1.0	1.0

* Fisher´s Exact Test

*n*: number of patients

### Risk factors for anastomotic leakage

**Figure 1.**
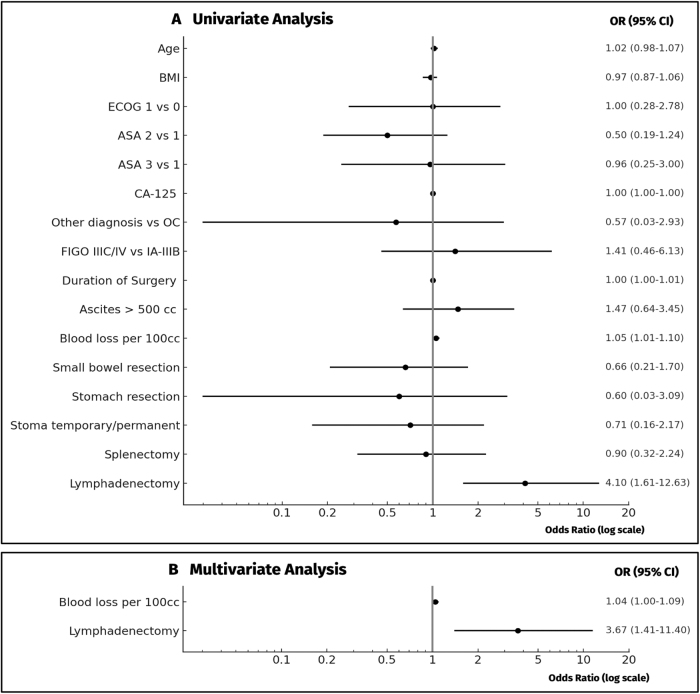
Risk factors for anastomotic leakage in univariate and multivariate analysis. Legend: OR: odds ratio, 95% CI: 95% confidence interval, OC: ovarian cancer, cc: cubic centimeter.

In univariate analysis, blood loss (OR 1.05 per 100cc; 95% CI 1.01–1.10) and lymphadenectomy (OR 4.10; 95% CI 1.61–12.63) were the only two characteristics identified as potential predictive factors for anastomotic leakage, while all other analyzed characteristics (age, BMI, ECOG, ASA physical status classification system, CA-125, cancer origin, FIGO, duration of surgery, ascites, small bowel resection, stomach resection, stoma placement, splenectomy) were not (Fig. 1). Both factors (blood loss [OR 1.04 per 100cc, 95% CI 1.0001–1.09], lymphadenectomy [OR 3.67, 95% CI 1.41–11.40]) were found to remain significant independent factors in step-back evaluation for multivariate analysis (Fig. 1).

Accordingly, blood loss and lymphadenectomy are parameters associated with anastomotic leakage rate. For all patients and the Non-lymphadenectomy subgroup, anastomotic leakage rate increased with higher blood loss, reaching the highest rates when blood loss exceeded 1500 cc (all patients: <500 cc: 5.8%, 500–1500 cc: 6.0%, >1500 cc: 14.5%, *P* = 0.11; non-lymphadenectomy: <500 cc: 0.0%, 500–1500 cc: 2.0%, >1500 cc: 13.6%, *P* = 0.03). In contrast, anastomotic leakage rate in the lymphadenectomy subgroup remains over 10% regardless of the amount of blood loss (<500 cc: 12.5%, 500–1500 cc: 10.2%, >1500 cc: 15.2%, *P* = 0.70) (Table 3).

**Table 3:** Number of patients with anastomotic leakage and rate according to the surgical procedures lymphadenectomy and blood loss

Blood loss	anastomotic leakage (rate) according to surgical procedure		
	All patients	lymphadenectomy	Non-lymphadenectomy
	(*n* = 323)	(*n* = 163)	(*n* = 160)
<500 cc	4/69 (5.8%)	4/32 (12.5%)	0/37 (0.0%)
500–1500 cc	12/199 (6.0%)	10/98 (10.2%)	2/101 (2.0%)
>1500 cc	8/55 (14.5%)	5/33 (15.2%)	3/22 (13.6%)
p-value*	0.11	0.70	0.03

* Fisher´s Exact Test

*n*: number of patients, cc: cubic centimeter

### Survival

**Figure 2.**
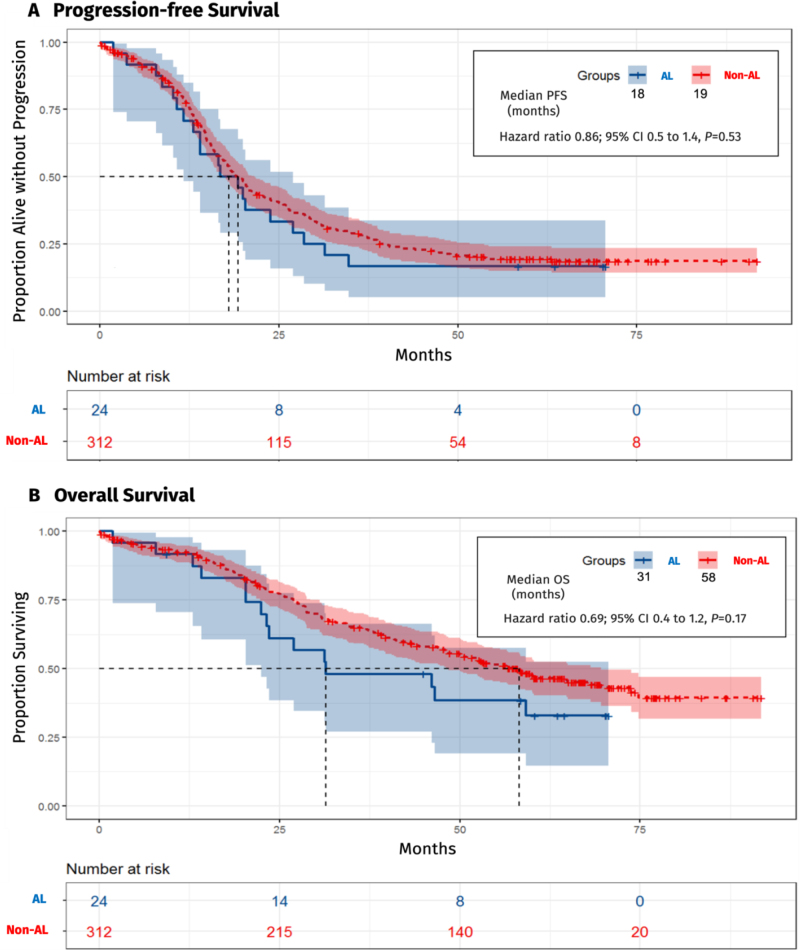
Progression-free survival and overall survival in patients with anastomotic leakage vs. Non-anastomotic leakage. Legend: AL: anastomotic leakage, PFS: progression-free survival, OS: overall survival, 95% CI: 95% confidence interval.

In Kaplan–Meier analysis, median PFS was comparable with 18 months in patients with anastomotic leakage vs. 19 months in the Non-anastomotic leakage group (hazard ratio (HR) 0.86; 95% CI 0.5 to 1.4, *P* = 0.53). Regarding OS, a median of 31 months was estimated in the anastomotic leakage group compared to 58 months in the Non-anastomotic leakage group, although the difference did not reach statistical significance (HR 0.69; 95% CI 0.4 to 1.2, *P* = 0.17) (Fig. 2).

## Discussion

This exploratory subgroup analysis of the prospective surgical phase III AGO-OVAR.OP3/LION trial with a homogeneously treated cohort of patients undergoing multivisceral surgery for ovarian cancer surgery underscores the critical importance of addressing anastomotic leakage as a serious perioperative complication following bowel surgery for advanced ovarian cancer. The significantly lower anastomotic leakage rate observed in the Non-lymphadenectomy subgroup with 3.0%, which represents the current standard of care, highlights the potential impact of reducing surgical complexity to minimize risk for anastomotic leakage. These findings emphasize the need to carefully balance the extent of surgical interventions with the associated risks of complications, particularly anastomotic leakage, to improve overall patient outcomes and ensure optimal perioperative management.

Several studies have provided compelling evidence of the significant and adverse effects of anastomotic leakage on the postoperative course of ovarian cancer patients, resulting in extended hospital stays, reduced likelihood of initiating chemotherapy or delays in the initiation of chemotherapy, which may have negative implications for patients’ overall survival^[6,9,11,12,14-17,24]^. In our analysis, while anastomotic leakage demonstrated a numerical trend toward worse outcomes for PFS and OS, these trends did not exhibit statistical significance. This distinction is critical because numerical differences alone are not sufficient to establish causal or prognostic associations. Nevertheless, these observations contribute to previously published data being ambivalent regarding a significant effect on postoperative mortality rate and OS^[9,11,14,23,24,34]^.

Currently, there is still a lack of identification of the specific population at the highest risk for anastomotic leakage, as well as established strategies to effectively prevent its occurrence. The assessment of anastomotic leakage risk factors in the ovarian cancer literature appears heterogeneous, potentially due to the rarity of anastomotic leakage and the individual surgical techniques used for intestinal resection and anastomosis^[6,9-12,14-17,19,22,24,25]^.

In our analysis, two independent factors, blood loss and lymphadenectomy were identified as potential risk factors for anastomotic leakage, while an impact of other documented clinicopathological characteristics could not be validated. Higher volumes of blood loss are associated with an increased rate of anastomotic leakage, particularly in the non-lymphadenectomy subgroup. In colorectal cancer surgery, former studies have proposed that intraoperative blood loss can be considered as an independent risk factor for anastomotic leakage^[35-38]^. Among the multiple factors involved in gastrointestinal anastomotic healing, guaranteeing continuous blood supply plays a central role in the development of anastomotic leakage, and the effect of ischemia on anastomotic healing disturbances is widely accepted. Therefore, meticulous surgical technique including precise dissection within tissue layers is crucial for ovarian cancer surgery to prevent large volume shifts. The use of indocyanine green fluorescence angiography may help to reduce risk for anastomotic leakage in bowel surgery and could become an approach in ovarian cancer surgery as well^[39]^. Additionally, timely intraoperative and anesthetic interventions to minimize blood loss, such as administering anti-fibrinolytic agents along with optimized preoperative patient management, play essential roles to improve patient outcomes and reducing complications^[40]^. Both the Enhanced Recovery After Surgery (ERAS®) Society and the European Society of Gynaecological Oncology (ESGO) are actively pursuing efforts to enhance the perioperative management protocols for patients diagnosed with ovarian cancer. This focus on optimization reflects a commitment to improving surgical outcomes and overall patient care. In line with these objectives, comprehensive guidelines have been developed and published, aiming to standardize and elevate the quality of care provided to this patient population^[41-44]^.

The observation that lymphadenectomy may be associated with a higher risk of anastomotic leakage in this exploratory analysis suggests several potential explanations that warrant further investigation. One possibility is that systematic pelvic and paraaortic lymphadenectomy increases the complexity and duration of surgery, as it involves meticulous dissection and removal of lymphatic tissue from the pelvic and paraaortic regions. Prolonged surgical time has been proposed to contribute to higher rates of postoperative complications, including anastomotic leakage, potentially due to cumulative tissue trauma, prolonged ischemia, and heightened inflammation^[45,46]^. Additionally, the extensive dissection required for lymphadenectomy might reduce the blood supply to adjacent bowel segments, which could impair anastomotic healing^[47]^. Another consideration is the potential impact of lymphatic disruption on the postoperative environment. Manipulation and removal of lymph nodes during lymphadenectomy may lead to lymph leakage or the accumulation of lymphatic fluid in the retroperitoneal space. This fluid, which is rich in inflammatory cytokines and proteins, might impair the healing of an anastomosis by promoting a local inflammatory milieu or increasing the risk of secondary infections^[48]^. While these hypotheses are plausible, further studies are needed to confirm these potential mechanisms.

As the primary analysis of AGO-OVAR.OP3/LION revealed that in advanced stage disease and in case of non-suspicious lymph nodes following complete cytoreduction, lymphadenectomy did not have a prognostic effect on neither OS nor on PFS, omission of lymphadenectomy has become standard of care as reflected in current guidelines. This may not only reduce surgical morbidity but also minimize the risk of complications like anastomotic leakage^[30,49,50]^. This finding reinforces the importance of critically evaluating the necessity of lymphadenectomy in surgical oncology, particularly in cases where it does not alter oncological outcomes. Surgeries performed in alignment with current recommendations for advanced ovarian cancer demonstrated acceptable rates of anastomotic leakage.

Our study did not find any statistically significant difference in anastomotic leakage rates among patients undergoing protective stoma placement and are in line with other published data^[24,51]^. To date, the decision-making process for performing a protective stoma placement is mostly based on individual intraoperative aspects and based on personal experience rather than objective risk factors for anastomotic leakage.

Contrary to the intended protective effect, stoma formation can also be associated with increased morbidity like dehydration, malnutrition and renal impairment due to increased outflow debit^[19]^. Furthermore, it can affect the patient’s self-image and has been associated with serious psychological effects and a decrease in quality of life^[52]^. Otherwise, protective stoma placement decreases the rates of overall complications such as sepsis and the need for re-laparotomy^[17]^. Thus, there is a group of patients, which certainly benefits from stoma placement.

Nevertheless, based on the present data, bowel surgery without stoma formation can be regarded as an appropriate approach regarding anastomotic leakage rate in advance ovarian cancer surgery as no significant differences and impact have been seen. Further studies are needed to better define this population and to make intraoperative decision-making as objective as possible.

Our study has several potential limitations. The study cohort of the AGO-OVAR.OP3/LION trial was not statistically powered for our exploratory analysis. Thus, we are aware that the limited number of anastomotic leakage events makes the risk factor analysis less reliable. Furthermore, we could analyze only a limited panel of clinicopathological characteristics, collected in AGO-OVAR.OP3/LION, and were not able to evaluate other former reported risk factors (i.e. surgical technique, distance from anal verge, additional blood tests like albumin levels)^[8-10,21-25]^. Moreover, analysis of risk factors associated with surgery like surgery duration or blood loss is quite complex due to missing standardized cut-offs. The potential strength of our study is the data quality as a subgroup analysis of a prospective phase III multicenter trial with surgery performed in high volume centers for ovarian cancer surgery defining a homogenous, well-treated cohort – a thorough database which has not been available for detailed analyses of surgical aspects so far.

## Conclusion

In conclusion, our subgroup analysis of the AGO-OVAR.OP3/LION trial indicates that anastomotic leakage rates in surgeries for advanced ovarian cancer appear acceptable when performed under the current standard surgical approach in high-volume centers. Bowel surgery without stoma formation can be considered as an appropriate approach regarding anastomotic leakage rate in these patients, although final decision has to be made based on individual intraoperative findings and patient-specific risk factors. In our analysis, anastomotic leakage demonstrated a numerical trend toward worse PFS and OS; however, these trends did not reach statistical significance. This underscores that anastomotic leakage remains a serious perioperative complication requiring meticulous postoperative patient care. The lack of statistical significance highlights the necessity of cautious interpretation of these findings and emphasizes the need for further research to validate these observations and develop targeted strategies for the prevention and management of anastomotic leakage.

Blood loss and lymphadenectomy, as potential surrogate markers for the extent of surgical intervention, should be carefully considered in the context of perioperative management for patients with advanced ovarian cancer. In contrast, no significant association was identified between anastomotic leakage and other evaluated clinicopathological characteristics in this analysis.

## Data Availability

Not applicable.

## Research registration unique identifying number (UIN)

LION ClinicalTrials.gov number, NCT00712218.

## Presentation

2023 ASCO Annual Meeting.

## Assistance with the study

We thank all AGO-OVAR.OP3/LION patients and trial investigators for participating in the LION trial and the according data collection.
